# Biological and Habitual Factors That Influence Sleep and Circadian Rhythm: A Narrative Review

**DOI:** 10.7759/cureus.113792

**Published:** 2026-08-01

**Authors:** Veronica J Harmon, Christopher J Davis

**Affiliations:** 1 Department of Translational Medicine and Physiology, Sleep and Performance Research Center and Gleason Institute for Neuroscience, Washington State University Elson S. Floyd College of Medicine, Spokane, USA

**Keywords:** biologic clock, chronotype, circadian misalignment, circadian rhythm, gut brain microbiome, modifiable sleep changes, peripheral oscillators, sleep, sleep quality & quantity, sleep-wake cycle

## Abstract

Sleep loss and circadian misalignment can adversely affect health. As such, a comprehensive understanding of what factors influence sleep loss and circadian misalignment benefits medical and scientific progress. While the interaction between light exposure and circadian entrainment and their relationship to circadian alignment is long-established, other factors that influence central or peripheral clocks (bodily oscillators) also contribute to circadian alignment and the potential for sleep loss. In this review, we will highlight the core factors that influence vulnerability or resilience to sleep loss and circadian misalignment, including biologic factors, such as gene haplotype, chronotype, and the gut microbiome. Furthermore, we will consider how habitual and largely modifiable variables, such as timing of meals and physical activity, can be manipulated to affect circadian and sleep cycles. Gaps in current scientific research on the effects of circadian misalignment and sleep loss will also be identified. This review will thus help inform future research and enhance the understanding of how healthy sleep and balanced circadian rhythm can improve quality of life.

## Introduction and background

Sleep is one of many bodily functions mediated by circadian rhythm. While recent sleep-wake history drives the homeostatic mechanisms of sleep need in the brain, the time-of-day governance of the sleep-wake cycle is regulated by the suprachiasmatic nucleus (SCN), an area of the anterior hypothalamus that consists of approximately 20,000 neurons and serves as a master timekeeper [1,2]. Ambient light stimulates specialized photoreceptive retinal ganglion cells at the back of the eye, where they detect the photic stimulus [2]. From there, the signal travels through the optic nerve to the SCN via the retinohypothalamic tract [3]. In this way, light is a powerful zeitgeber that entrains the SCN to communicate circadian oscillations to other systems in the body to help regulate sleep-wake cycles [3]. However, it is not light alone that influences sleep propensity and SCN rhythmicity. Peripheral oscillators, which are located in various organs and tissues throughout the body, can also operate independently through their own metabolic mechanisms and can function in tandem or as feedback loops with the central SCN to regulate circadian rhythms and sleep/wake patterns [2]. Disturbances to this complex pathway can lead to circadian misalignment or disruption and can inherently impact sleep metrics and overall health [2-4].

Several metrics are employed to determine the degree to which an individual experiences sleep loss. Measurable terms are defined to assess the multifaceted nature of sleep and its objective and subjective qualities. Sleep duration quantifies the total amount of time someone has slept in a 24-hour period. Sleep continuity evaluates the ability to fall and stay asleep. Sleep quality is a subjective measure of whether one would regard their own sleep as satisfying. Sleep depth is objectively analyzed by polysomnography (PSG) to quantify concurrent brain and muscle activity, with decreasing values potentially indicative of sleep loss [4]. Cortical brain waves can also be probed to characterize the electroencephalograph (EEG) spectral content, including the activities of the delta, theta, alpha, beta, and gamma spectral windows, as well as slow-wave sleep (SWS) and rapid eye movement (REM) sleep [4,5]. In addition, actigraphy is often used to measure behavior and movement during wake and sleep [4]. 

The positive effects of adequate sleep on cardiovascular, cerebrovascular, and immune health as well as mood and cognitive function are well-documented [6,7]. Conversely, the short-term effects of inadequate sleep include cognitive and performance deficits as well as increased activation of the stress-driven sympathetic nervous system [8]. The long-term effects of chronic sleep insufficiency include increased risk for cardiovascular disease, such as associations with myocardial infarctions, as well as metabolic diseases [8]. Despite these well-studied implications, approximately one-third of adults do not get the recommended seven to eight hours of sleep in a 24-hour period [7]. This knowledge has propelled research into characterizing the variables that determine vulnerability or resilience to sleep loss and circadian misalignment to improve the overall health of the public and ward off increased risk of the aforementioned ailments [9,10]. 

In this review, we will analyze biological as well as practical, modifiable factors and their propensity to provide resilience against or contribute to the vulnerabilities of sleep loss or circadian misalignment. Specifically, chronotype, gene haplotype, and the gut microbiome are biological factors that play important roles in governing sleep/wake cycles and circadian rhythms [5,9-19]. We will also consider modifiable, habitual cues, such as the timing of dietary consumption and physical activity, for their potential to provide protection against sleep and circadian rhythm disruptions [20-25]. Discussion of these variables serves to provide a brief rendition of what risk factors endanger sleep and circadian health and may guide research that will advance our understanding of sleep; the byproduct of increased research on sleep science and clinical application thereof can help inform the diagnosis and treatment of sleep disorders and circadian misalignment.

This review primarily implemented a literature search using PubMed. Search terms included keywords and combinations of keywords related to circadian rhythm, circadian misalignment, sleep loss, chronotype, gut-brain axis, microbiome, meal timing, and physical activity. Formal publication date parameters were not applied; however, priority was given to the more recently peer-reviewed literature. Articles were selected based on their relevance with regard to factors that influence sleep loss and circadian misalignment. As this was a narrative review, no formal meta-analysis was performed, and no cross-study quantitative synthesis was derived. 

## Review

Individual biology and circadian rhythm 

Chronotype

Chronotype reflects the preferential tendency of an individual’s circadian clock, including but not limited to their sleep-wake cycle, to peak earlier or later in a 24-hour period [9,11,26]. Chronotype can shift during an individual’s lifetime, with adolescents typically undergoing a phase delay and elderly people experiencing a phase advance [9,26]. Tools like the Morningness-Eveningness Questionnaire (MEQ) categorize people into early, late, or intermediate chronotypes [9,27]. Late chronotypes, or ‘owls,’ are triggered via their internal circadian clocks to sleep and wake up later in the 24-hour period; however, for early chronotypes, sometimes referred to as ‘larks,’ these signals occur at earlier times [9,26]. As such, sleep pressure builds up earlier in the day for early chronotypes than their late or intermediate chronotype counterparts.

Expectations from modern-day society can clash with chronotype [9,26]. People who work in medicine or construction or various other fields may be required to work overnight and thus may experience misalignment of their circadian rhythm [8,26]. This is particularly true for early chronotypes. In contrast, late chronotypes may experience conflicts between their circadian rhythm and what are considered societal waking hours, the operating hours of typical businesses and most public schools [9,26]. Therefore, simply due to the demands of modern society, including early work and school hours, traffic, or daylight, late chronotypes may be more vulnerable to misalignment of their sleep-wake circadian cycle, and thus experience more sleep curtailment than earlier chronotypes in those circumstances [9]. This misalignment of chronotype-influenced circadian clocks and sleep-wake cycle is termed internal desynchronization, which can, ultimately, lead to increased cumulative sleep loss and heightened risk of adverse health effects [11]. 

Although there are many studies reporting that certain chronotypes have difficulty functioning optimally in a society with demanding and unsupportive sleep schedules (e.g., early start times, graveyard shifts, and fluctuating schedules for larks compared to owls) [9,26], there are few that identify which chronotypes are more susceptible or vulnerable to the effects of sleep loss. The propensity to be vulnerable to sleep loss due to circadian preference has been measured with a post-sleep restriction comparison of chronotypes. Cox et al.'s study showed that late chronotypes experience more negative affect (higher frequencies of emotions like anger, anxiety, and sadness) after sleep restriction than their earlier chronotype counterparts [9]. These negative emotional manifestations have been associated with poor physical and mental health outcomes [28]; however, further research would be indicated to propose a potential association between sleep-deprived late chronotypes and the negative consequences of sleep deprivation.

Gene Haplotype 

Certain genetic biomarkers and haplotypes are associated with sleep loss and circadian misalignment via various downstream biochemical consequences [5,10,11,29]. *PER3* (Period Circadian Clock 3) is a clock gene on human chromosome 1 [5,11]. Variation in the number of tandem repeated segments (either 4 or 5 units) on *PER3* may lead to alternate posttranslational modifications, which affect sleep by modulating circadian phase or the expression of SWS [5]. Studies have shown that people homozygous for *PER3* 5/5 fall asleep more quickly and demonstrate more SWS, resulting in less sleep loss and greater sleep depth than carriers of *PER3* 4/4 [5]. SWS is characterized by high-amplitude waves in the 0.5-4 Hz range of the non-REM (NREM) sleep EEG and represents the deepest stages (stage N3) of NREM sleep, often characterized as restorative sleep [5,30]. Faster sleep onset and more time in deep sleep could result in more sleep and improved sleep quality [5]. Furthermore, variations of *PER3* have been shown to correlate with chronotypes, with *PER3* 5/5 displaying an association with morning-types (larks) and *PER3* 4/4 associating with evening-types (owls) [11]. Interestingly, when carriers of *PER3* 4/4 were compared to carriers of *PER3* 5/5 after exposure to total sleep deprivation, the effects of sleep deprivation in *PER3* 5/5 included diminished executive and cognitive function in the early morning and reduced attention [10]. The dissonance in the protective mechanism against total sleep loss (falling asleep more quickly and demonstrating more SWS) compared to the seeming vulnerability to experience the effects of sleep loss (diminished executive and cognitive function) for *PER3* 5/5 carriers is a finding that warrants further studies on these and other haplotypes. 

In addition to *PER3*, a variant of brain-derived neurotrophic factor (BDNF), a key neurotrophin in the brain that supports neural development as well as plasticity, has also been associated with the response to sleep loss [10]. More specifically, a single nucleotide substitution in the *BDNF* gene results in an amino acid change from valine to methionine at position 66 (Val66Met) that has been associated with decreased propensity to withstand sleep deprivation compared to its Val66Val counterpart. After 20 hours of sleep deprivation, individuals with the Val66Val outperformed those with the Val66Met variant on the Stroop Test, which measures cognitive control and adaptability [10,31]. After 40 hours of sleep deprivation, Val66Val carriers also performed better than Val66Met heterozygotes on a memory task. However, when vulnerability to the effects of circadian misalignment was analyzed, the resilience to misalignment shifted in favor of the Val66Met polymorphism. Val66Met heterozygotes were able to complete psychomotor vigilance tasks (PVTs) with fewer lapses, a reflection of cognition and performance less impacted by circadian misalignment. Their sleep EEGs also showed less SWS and lower delta and theta frequencies in NREM sleep at baseline as well as after 40 hours of sleep deprivation [10]. Overall, people with the Val66Met *BDNF* variant seem to withstand the effects of circadian misalignment better than those with its homozygous Val66Val counterpart but are more susceptible to the effects of sleep loss [5,10]. Whether or not this observation is due to the general reduction in SWS and lower delta and theta frequencies during NREM sleep or another factor in carriers of the Val66Met variant should be explored in future investigations. 

Genetic variations have also been associated with sleep anomalies [10,29]. For example, the *BHLHE41 (*basic helix-loop-helix family member e41) gene, also known as *DEC2*, has been shown to modulate circadian rhythm and sleep [29]. A mutation in the *DEC2* gene that results in an amino acid change from proline to arginine at position 284 has been associated with familial natural short sleep [10]. These short sleepers are less susceptible to sleep loss, as their homeostatic baseline requires less sleep. In addition, despite spending less time asleep, they display less nighttime serum melatonin and an earlier peak in cortisol in the morning [29]. In contrast, the human leukocyte antigen (HLA) DQB1*0602 allele has been associated with narcolepsy, with individuals who are positive for HLA DQB1*0602 reporting more subjective fatigue after sleep restriction [10]. These findings underscore the need to distinguish genetic influences on sleep loss versus circadian misalignment, as they represent distinct physiological challenges.

Identifying genetic markers related to sleep loss vulnerability or resilience is helpful for advancing sleep science, yet there are current limitations to what we know that fuel further discovery efforts. For instance, there are discrepancies in the results of genetic variant studies regarding susceptibility to the effects of sleep loss versus the effects of circadian misalignment [10,29]. Although these effects may seem complementary to one another, they are applicable to different situations, i.e., sleep deprivation due to insomnia versus circadian misalignment due to shift work, and thus affect individuals differently. Having a clear understanding of what conditions affect which element of sleep can help focus the development of and usage of pharmacological and non-pharmacological therapies targeted towards better sleep and overall health [7,10]. 

Gut Microbiome 

Unlike genetic factors, which are largely inaccessible, individualized data points that are, currently, mostly unmodifiable, the gut microbiome represents a biologic variable that is more observable and potentially modifiable [12,13]. The gut microbiome is the community of microbes, including bacteria and other microorganisms, that reside in the gut [12-14]. A typical human gut microbiome consists of hundreds of types of bacteria, including but not limited to *Firmicutes*, *Bacteroides*, *Cyanobacteria*, *Fusobacteria*, *Verrucomicrobia*, *Proteobacteria*, and *Actinobacteria *[12,13].

In humans, the initial colonization and development of the gut microbiome is shaped by various environmental factors during the first three years of life, with the microbiota being continually influenced by external factors, including diet, stress, and medications [12]. For example, certain phyla, namely, *Firmicutes* and *Bacteroides*, have been shown to fluctuate in quantity in accordance with the quality and frequency of nutrition intake [13], suggesting that the composition of the microbiome is modifiable. The composition of the microbiome is important because it can have significant effects on the physiological systems of the host, such as metabolism and the immune system [13,14,17]. The implications of a healthy or unhealthy gut microbiota are not unidirectional in their influence on circadian misalignment resilience or susceptibility; rather, there is a cyclical relationship [12-14]. Furthermore, the microbes themselves display qualities that would imply they have their own circadian rhythm [13,14].

The gut and its microbial components communicate with the brain via the microbiome-gut-brain (MGB) axis or the gut-brain-axis (GBA). Three distinct pathways, the neuroendocrine, immunoregulatory, and vagus nerve pathways, allow the microbiota to communicate with the brain in a bidirectional fashion, resulting in a multitude of effects that correspond with sleep health [13]. In the neuroendocrine pathway, neuroendocrine cells in the gastrointestinal tract called enteroendocrine cells (EECs) secrete neurotransmitters, such as gamma-aminobutyric acid (GABA) and serotonin, similar to neurons in the brain. It is postulated that these cells can sense the make-up of the microbiota and modulate their own secretions and actions accordingly [14,15]. Various species of gut bacteria can also produce their own neurotransmitters and hormones, namely norepinephrine and dopamine as well as serotonin, melatonin, and cortisol, which affect the brain and homeostasis via various feedback loops and response mechanisms [13,14,16].

In the immunoregulatory pathway, communication in the MGB axis is determined by the bacteria and their metabolites in the gut that affect immune cells and their actions in the body. For example, some bacteria elicit pro-inflammatory responses from immune cells [13,17]. When the gut is in eubiosis, regulatory T cells produce anti-inflammatory cytokines [14,17]. These anti-inflammatory mechanisms can seep into the circulation and promote anti-inflammatory effects to the rest of the body as well as promote healing and prevent disease. By contrast, dysbiosis in the gut, a microbial imbalance that can lead to loss of the integrity of the intestinal epithelial barrier, can promote a pro-inflammatory state [14,17]. A decrease in the integrity of the gastrointestinal lumen allows inflammation to spread to the periphery, causing further damage and increasing the risk of metabolic disease. This relationship is bidirectional in that damage to the brain can cause a pro-inflammatory response which can reach the gut, compromising its permeability and allowing bacterial contents from the lumen to enter the circulation [14].

Finally, in the vagal pathway or more broadly, neuroanatomic signaling, the vagus nerve functions as a communication highway between the brain and the gut microbiota [14]. Consequences of this communication can include the transmission of neurotoxic bacterial metabolites to the central nervous system (CNS), negatively affecting neural function, including sleep health [13]. The enteric nervous system (ENS) in the intestines, which controls peristalsis as a function of the autonomic nervous system, also communicates with the vagus nerve. The gut microbiome is in contact with sensory neurons of the ENS, providing indirect communication to the vagus nerve and thus the brain [13,14]. 

The gut microbiome and its communication with the rest of the body relates to sleep and one’s vulnerability to sleep loss or circadian misalignment through various mechanisms. One way is via communication between the gastrointestinal tract and other physiologic systems through the neuroendocrine pathway. This pathway is mostly regulated by neurotransmitters emitted by neuroendocrine cells, influenced by bacteria, affecting the brain’s circadian rhythm and sleep drive [13-16]. For instance, lower levels of the neurotransmitter GABA, due to deficiencies in the bacteria *Bifidobacterium* and *Lactobacillus*, can increase vulnerability to sleep loss [16]. The majority of the serotonin produced in the body is secreted by neuroendocrine cells, specifically enterochromaffin cells that are housed within the gastrointestinal tract, as well as by certain bacteria that live in the gut, such as *Enterococcus* and *Escherichia* *coli* [13]. Enterally produced serotonin does not cross the blood-brain barrier; however, both serotonin in the CNS and peripheral serotonin require tryptophan as precursors [13,14,16]. Dysbiosis in the gastrointestinal system can cause inflammation, which activates enzymes that convert a substantial amount of systemic tryptophan into kynurenine [14]. This leaves less tryptophan available to be converted into serotonin both in the CNS and peripherally. Serotonin plays an important role in REM sleep and is a precursor to melatonin, which regulates sleep-wake cycles [13,17]. Moreover, due to the indirect connection between the gut and the brain via the vagus nerve, neurotoxic byproducts produced by intestinal bacteria, such as D-lactic acid and ammonia, can interfere with sleep quality and increase one’s susceptibility to sleep loss [16]. 

Another way that systemic communication with the microbiome affects sleep health is through the immunoregulatory pathway, which mediates the cyclical relationship between inflammation and the sleep response [14,16,18]. Not getting enough sleep can lead to inflammation in the body [6,18]. Bacteria in the gut stimulate immune cells to increase the levels of cytokines, such as tumor necrosis factor alpha, interleukin (IL)-1 beta, and IL-6, as well as C-reactive protein and prostaglandins to elicit an immune response [14,16]. Sleep loss can also make one more vulnerable to infection by way of oxidative stress that damages the tight junctions between intestinal epithelial cells, allowing bacteria to enter the systemic circulation [14]. Conversely, inflammation or an immune response due to infection can promote sleep [18]. For example, when lipopolysaccharides, which are molecules found on the outer membrane of gram-negative bacteria, elicit an immune response, the cytokines produced bind to receptors on the afferent vagus nerve. This sends signals directly to the CNS, creating a somnogenic effect. Although inflammation can increase the drive and need for sleep, it is not a protective factor against sleep loss [18]. Rather, the induction of sleep due to inflammation is a response mechanism to promote fever and thus kill the potential microbes that led to the initial inflammation. In addition, the pro-inflammatory cytokine IL-1 is thought to increase the SWS phases of NREM [18]. 

Exactly what the makeup of the microbiota must be to protect individuals from sleep loss or circadian misalignment is still unclear [16,19]. Limited emerging research points to the strains *Lentisphmulaeria*, *Victivallaceae*, *Lentisphaerae*, *Odoribacter*, and *Lachnospiraceae* as being associated with longer sleep duration, alluding to reduced vulnerability to sleep loss. Conversely, higher levels of *Selenomonadales* and *Negativicutes* may put individuals at higher risk of suffering from insomnia, consequently increasing susceptibility to sleep loss [16]. Specific bacteria can also influence the chronotype of individuals. Evening-type individuals were found to have higher levels of *Enterobacteriales* and *Enterobacteriaceae* in their gut than morning-types [19]. Therefore, we must ask whether our chronotype can be changed by changing the composition of our gut microbiota. To answer this question, further research is warranted to determine whether significant changes in chronotype can be achieved via dietary changes or direct transplantation of gut microbiota to influence gut bacterial composition. 

Studies analyzing germ-free mice (mice that have never had functional or effective microbiota) have found that these mice have reduced expression of circadian clock genes in their SCN, diminishing their functional oscillators. This finding promotes the notion that the microbiome is a key contributor to the efficacy of clock gene expression [13]. Conversely, bacteria, specifically motile gram-negative species in the gastrointestinal tract, activate the transcription factor nuclear factor IL-3 (NFIL3), which is known to be involved in regulating circadian rhythms [14]. The gut microbiota can also affect host circadian clock genes by modulating their expression or by regulating their downstream effectors and byproducts, which then affect the chronotype and circadian rhythm of the host [13,14,17]. Moreover, some gut microbes exhibit their own diurnal oscillators which, under varying influences, affect their production of short-chain fatty acids, metabolites, and byproducts and their regulation of hormones. This oscillation is greatly determined by environmental cues, mostly modifiable by the human host [17]. Bacteria, namely *Bacteroides* and *Firmicutes* species, produce short-chain fatty acids (SCFAs) such as butyrate, acetate, and propionate as metabolic byproducts of dietary fiber. Although the exact mechanism is unclear, in mice, SCFAs seem to decrease wake time and increase NREM sleep [16,32]. It has also been proposed that the anti-inflammatory effects of SCFAs, and their promotion of metabolic homeostasis and decreased gut permeability, are protective against sleep loss and circadian misalignment [16,17,32]. 

Research has highlighted the negative impacts that poor sleep can have on the gut microbiota and its sequelae, including bacterial overgrowth, increased gut permeability, and increased risk of metabolic disease [13,14,16,32]. However, the gut microbiome’s effects on sleep and vulnerability to sleep loss are not as extensively studied. There remains ubiquitous rhetoric regarding the cyclical relationship between sleep and the gut, with poor sleep manifesting as poor gut health that, in turn, leads back to poor sleep [13,14,32]. The exact parameters of a healthy gut continue to be ambiguous, making it difficult for clinicians to provide specific guidance to patients with poor sleep health [16,32]. Thus, more research on the composition of a healthy gut microbiome and its effect on vulnerability to sleep loss or circadian misalignment could yield clinical benefit; much of the research on the matter lacks conclusiveness because of the scarcity of research [14,32]. However, it could be argued that a generalizable, healthy, and diverse microbiota is sufficient to positively impact sleep health and metabolism, and that knowing the exact species of bacteria that are present in the gut is a nuance that might be too variable (particularly due to the daily changes in the composition and activity of the microbiome) to enable the development of any specific composition recommendations at this time [16]. 

Diet, physical activity, and sleep 

It is widely accepted that our daily habits contribute to our overall wellbeing. With seemingly endless information readily available at our fingertips, the internet can provide numerous potential methods to live a healthy lifestyle. Health and how to optimize it have also been the subject of scientific study for years [4,7]. The time of day and duration of eating, as well as the intensity and length of activities undertaken, seem to contribute not only to how we live our lives while awake but are also suggested to be modifiable factors in one’s sleep health [16,24,25,33].

Dietary Consumption 

The time of day that individuals eat seemingly affects their circadian rhythm [16,33,34]. Typically, under normal diurnal conditions, the human body is most active between the hours of 10:00 AM and 10:00 PM. The ingestion of food affects hormone secretion and metabolism and thus impacts peripheral rhythm [16]. Peripheral oscillators that reside in the liver, gastrointestinal tract, and pancreas are not exposed to light. Therefore, these oscillators depend on the influence of hormones and signals that are a result of the intake and digestion of food to recognize which phase the body is in during its 24-hour cycle, as humans are typically awake while they eat [17,34]. When food is available, its resulting signals dominate the circadian control of the peripheral oscillators, operating as a zeitgeber [33,34]. However, in the absence of conflicting feeding cues, peripheral oscillators adapt to the rhythm dictated by the SCN [2,33]. Therefore, as the SCN responds to natural light and is biologically cognizant of the typical daylight hours, it is most advantageous to eat in congruence with the time of day in which the central oscillators believe the body is in its active and awake phase [2,33]. In other words, eating earlier in the day aligns better with the central clock if one has a diurnal schedule, while eating later in the evening disrupts SCN regulation and has been shown to pose metabolic effects, such as increased glucose and insulin levels and increased levels of C-reactive protein [33]. Therefore, misaligned meal timing can disrupt the synchrony between peripheral clocks and the SCN (determined by light), creating chronodisruption in sleep [34]. 

The anecdote that one should not eat a heavy meal before bed may hold merit, as the proximity of time between your last meal and the onset of sleep seems to be associated with increased wake after sleep onset (WASO). WASO refers to the accumulated quantity of time that a person is awake after the initial onset of sleep. A high WASO is characteristic of insomnia. Eating less than one hour before bed is associated with a higher WASO, making one more susceptible to sleep loss [35]. This is likely due, in part, to increased hypothalamic-pituitary-adrenal activity, particularly with calorically dense and slowly digestible foods [20]. Therefore, for healthy individuals, consuming the last meal of the day approximately four to six hours before the onset of sleep may be more protective against sleep loss [35]. 

On the other hand, it has been suggested that carbohydrate-dense foods before bed can possibly promote sleep. Carbohydrates signal the release of insulin, and increased insulin promotes the uptake of amino acids into the muscle, excluding tryptophan. The resultant tryptophan-rich serum theoretically allows more tryptophan to cross the blood-brain barrier than when there is a higher ratio of other amino acids in the serum. In the brain, tryptophan is converted to serotonin via tryptophan hydroxylase [21]. Serotonin induces a relaxed state for the individual and allows for an easier transition into sleep. Furthermore, serotonin is a precursor of melatonin [21,22], which binds to MT1 and MT2 receptors in the SCN with resultant sleep-promoting effects [23]. However, this theory has been challenged by evidence showing that a highly packed carbohydrate meal (carbohydrates making up 70% of the meal) nullified increased serum tryptophan levels if even 5% of the meal was protein-based [21]. Macronutrients are typically not consumed in isolation, and even with a small addition of protein, the somnogenic effect of carbohydrates becomes negligible [21]. In other words, the effect of carbohydrate-dense meals as a protective mechanism against sleep loss is likely not practical or sustainable outside of a highly controlled study. 

Physical Activity 

Skeletal muscle is yet another component that influences circadian rhythm [24,36]. Aerobic and anaerobic exercise are widely accepted to have positive impacts on sleep and health [24,25]. Accordingly, it is recommended that most adults participate in at least 150 minutes of moderate intensity or 75 minutes of vigorous physical activity per week [24]. Furthermore, regular, consistent exercise (defined by a range of time intervals) has been found to increase total sleep time and improve subjective sleep quality, reducing the vulnerability to sleep loss [25]. 

Scientific studies have not unanimously concluded whether routine morning or evening physical activity is inherently better for sleep and circadian alignment [24,25,36]. There is a physiologic phenomenon referred to as “dipping” in which the body responds to the sleep state by lowering blood pressure by approximately 10-20% compared to the waking state. It has been demonstrated that in people whose blood pressure does not normally dip at night, evening exercise was able to lower their blood pressure more than morning exercise. This result implies that evening physical activity could be more beneficial to those whose blood pressure does not dip before sleep, as is considered the normal physiologic pattern, but replications of similar methods have exemplified that morning exercise can also decrease nocturnal blood pressure [36]. The notion that evening exercise leaves individuals too stimulated for sleep has been challenged. Evening exercise in young adults and children without sleep disorders has been shown to improve sleep onset latency [24,25,36]. However, core body temperature (CBT) changes due to evening physical activity can affect sleep. Similar to systemic blood pressure, an appropriate decrease in CBT is optimal for sleep health, specifically, sleep onset [24]. Although physical activity can initially increase CBT (depending on the degree of vigor and duration of the activity), the compensatory vasodilation in the periphery can result in rapid lowering of CBT, facilitating sleep onset [25]. Therefore, it can be inferred that regular exercise before sleep may lower CBT following the post-exercise cooling response, thus promoting the onset of sleep and potentially reducing the risk of insufficient sleep. Ultimately, regular and consistent physical activity, irrespective of the time of day, is conducive to sleep health as long as it is not at the cost of total sleep duration [25]. 

It has consistently been concluded that the benefit of morning versus evening exercise on sleep quality and duration as well as circadian rhythm is largely individualized [24,25,36]. However, one factor that can help settle the ambiguity is the assessment of individual chronotype. Sleep and circadian rhythm may benefit from the alignment of physical activity in accordance with one’s lark or owl tendencies, optimizing their skeletal muscle performance to their chronotype. Early chronotypes may see misalignment of their circadian rhythm and thus a total reduction in sleep if they exercise late into the evening, while late chronotypes may see similar effects with early morning exercise [36]. 

The panacea-like benefits of physical activity on general health may overshadow or entangle the more subtle effects of exercise on sleep and circadian alignment. Therefore, it is difficult to pinpoint exercise as the direct cause for the various improvements in sleep health. Although this overlap may ultimately benefit individuals clinically, from a science and research perspective, it is difficult to determine whether the sleep improvements are due to the indirect benefits of physical activity, such as better metabolic or respiratory function or improved mood, or whether they are directly related to positive effects on circadian rhythm. Future research trials could clarify this distinction by isolating the positive health effects of physical activity apart from sleep improvements to gain a deeper and more specific understanding of these mechanisms.

## Conclusions

The physical and mental implications of sleep cannot be overstated. In alignment with the importance of sleep, the mediators that influence the resilience or vulnerabilities to sleep loss or circadian misalignment have been outlined. The less modifiable biological factors, such as gene haplotype, chronotype, and, to some extent, gut microbiome, must be considered so that those who are predisposed to suboptimal sleep and circadian patterns are aware of their impact and can help guide appropriate strategies to optimize sleep metrics. Habitual factors, such as meal timing and physical activity, in relation to their effects on circadian rhythm and sleep, can also be important measures in optimizing sleep strategies. Exploration of these factors and advancements in research thereof equip clinicians with the vital information needed to support their patients to improve their sleep as well as empower individuals to make informed decisions regarding their sleep and circadian alignment to optimize overall wellbeing and quality of life. Future research is warranted to distinguish direct versus indirect relationships between modifiable factors that influence sleep and gain a keen understanding of the precise steps that can be taken to improve sleep.
